# Detection of Horizontal Gene Transfers from Phylogenetic Comparisons

**DOI:** 10.1155/2012/813015

**Published:** 2012-05-23

**Authors:** Victor Satler Pylro, Luciano de Souza Vespoli, Gabriela Frois Duarte, Karla Suemy Clemente Yotoko

**Affiliations:** ^1^Laboratório de Biodiversidade e Biotecnologia para o Meio Ambiente, Departamento de Microbiologia, Universidade Federal de Viçosa, Avenida P.H. Rolfs, s/n, Viçosa, MG, Brazil; ^2^Laboratório de Biotecnologia, CCTA, Universidade Estadual do Norte Fluminense Darcy Ribeiro, Avenida Alberto Lamego, 2000, Parque Califórnia, Campos dos Goytacazes, RJ, Brazil; ^3^Centro Universitário Newton Paiva, Escola de Engenharia e Arquitetura, Rua José Cláudio Rezende, 420, Estoril, CEP 30494-230 Belo Horizonte, MG, Brazil; ^4^Laboratório de Bioinformática e Evolução, Universidade Federal de Viçosa, Avenida P.H. Rolfs, s/n, Viçosa, MG, Brazil

## Abstract

Bacterial phylogenies have become one of the most important challenges for microbial ecology. This field started in the mid-1970s with the aim of using the sequence of the small subunit ribosomal RNA (16S) tool to infer bacterial phylogenies. Phylogenetic hypotheses based on other sequences usually give conflicting topologies that reveal different evolutionary histories, which in some cases may be the result of horizontal gene transfer events. Currently, one of the major goals of molecular biology is to understand the role that horizontal gene transfer plays in species adaptation and evolution. In this work, we compared the phylogenetic tree based on 16S with the tree based on *dsz*C, a gene involved in the cleavage of carbon-sulfur bonds. Bacteria of several genera perform this survival task when living in environments lacking free mineral sulfur. The biochemical pathway of the desulphurization process was extensively studied due to its economic importance, since this step is expensive and indispensable in fuel production. Our results clearly show that horizontal gene transfer events could be detected using common phylogenetic methods with gene sequences obtained from public sequence databases.

## 1. Introduction

The discussion concerning bacteria phylogenies has become one of the most important aspects of microbial ecology. In the mid-1970s, Woese and his collaborators proposed and began assembling a significant database of sequence information based on small subunit ribosomal RNA (SSU rRNA 16S). The current universal tree is based on this [1–6], since it is easily sequenced (±1,500 nucleotides) and widely available in sequence databases (Gen-Bank, EMBL) [7, 8]. However, phylogenetic hypotheses based on several other genes result in conflicting topologies and reveal different evolutionary histories. In many cases, especially within bacteria, these may be the result of horizontal gene transfers (HGTs) [9, 10], which are regarded as a crucial mechanism of increasing genetic variability among bacteria [11–13]. Currently, one of the major goals of molecular biology is to understand the role that HGT plays in species adaptation and evolution [10, 14, 15]. The presence of HGT in bacteria has been reported for several years, suggesting that for some genes the tree of life becomes a net [16]. HGT is dominant among various groups of genes in prokaryotes such as antibiotic resistance, carbon source utilization, organic contaminant degradation, and freeze tolerance genes [12, 13]. However, there is some evidence of HGT in housekeeping genes such as those for replication, transcription, and translation as well [10, 17, 18].

Sulphur is the third most abundant element in petroleum (after carbon and hydrogen), and its release contributes to air pollution by causing acid rain [19, 20]. For this reason, sulphur regulations have continued to become more stringent and it is necessary to remove sulphur oxides from fossil fuels during the refining process. Most inorganic and simple organic sulphur can be removed by hydrodesulphurization, the technique currently used by most petroleum refineries, but, in petroleum, the majority of sulphur is found in dibenzothiophene (DBT) and its derivatives, which can only be removed through a specific biological mechanism called biodesulphurization [21]. Several studies have investigated the development of aerobic microbial desulphurisation pathways [22–24].

Some bacteria can desulphurize DBT to 2-hydroxybiphenyl (2-HBP) through the sulphur-specific degradation pathway (4S pathway) without destroying the hydrocarbon skeleton [22, 24–26]. In natural environments, the cleavage of carbon-sulfur bonds in molecules such as DBT liberates sulfur, making it available as a nutrient to support the growth of bacteria in environments poor in mineral sulfur [27]. These bacteria have been assigned to a number of genera including *Rhodococcus *[21, 28], *Acinetobacter*, and *Pseudomonas *[24]. Species of other bacteria genera, such as *Brevibacterium* sp. strain DO [29], strains identified as *Arthrobacter* spp. [30], and *Gordonia* sp. strain CYKS1 [31], are also able to use this pathway.

The pSOX plasmid [28], or *dsz* genes [32, 33], responsible for the sulfur oxidation in DBT, have been cloned, sequenced, and studied, generating considerable knowledge of these pathway enzymes [28, 32, 34]. The 4S pathway consists of three genes designated *dsz* A, B, and C. Studies have shown that the product of *dsz*C directly converts DBT to DBTO2 and the products of *dsz*A and *dsz*B act together to convert DBTO2 to 2-HBP. The operon *dsz* occupies a 4 kb gene locus in a 120 kb linear plasmid in bacteria *Rhodococcus erythropolis* strain IGTS8 [28, 32, 35–37]. The plasmid nature of the *dsz* genes increases the probability of successful transfers, and the availability of the *dsz*C sequences in GenBank allows the construction of phylogenetic hypothesis based on this gene, in order to compare it with the 16S.

In this work, we aim to demonstrate the utility of phylogenetic methods based on molecular data to help in studies of horizontal transfer of functional genes in bacteria.

## 2. Materials and Methods

### 2.1. Nucleotide Sequences

The nucleotide sequences used in this study were obtained from the National Center for Biotechnology Information-GenBank (http://www.ncbi.nlm.nih.gov). For analyses involving the *dsz*C gene, 18 sequences were selected (Table 1), representing all genera and/or species with *dsz*C sequences available in the database as of March 2012. We also searched for the other two genes of the operon, *dsz*A and *dsz*B, but they are underrepresented in GenBank and phylogenetic trees could not be constructed based on these genes. For the 16S gene, we chose 39 sequences, including at least two sequences of at least 1400 bp from each genera and/or species in the *dsz*C tree (Table 2).

### 2.2. Phylogenetic Analysis

Phylogenetic analyses were performed with four different methods: neighbour joining (NJ) using the program MEGA 5.0 [38]; maximum parsimony (MP) and maximum likelihood (ML) using the program PAUP* [39]; Bayesian inference (BA) using the program MrBayes [40]. For NJ, ML, and BA, we chose the best nucleotide substitution model using the programs Mega 5.0 [38], ModelTest [41], and MrModelTest [42]. The chosen models are shown in Table 3. We used the Tree Bisection and Reconnection heuristic search method to search for the MP and ML trees. The MP tree started with a random tree, while the ML tree started with an NJ tree. To infer the tree through the BA, we run two independent analyses with four chains each (one cold and three hot chains), started with four different random trees modified through 5,000,000 generations of MCMC. We checked the likelihood of the resulting topologies and burned-out 25% of the trees (to keep those within the area of the best likelihoods) to construct the consensus tree. The robustness of each node of the tree was obtained by the bootstrap test (MV, MP, and NJ); the posterior probability was calculated by the frequency of each node in the consensus BA tree.

### 2.3. Phylogenetic Network Estimation of *dszC* Genes

Given the phylogenetic hypothesis for the *dsz*C gene, we constructed a network using the most related haplotypes with statistical-parsimony analyses [43]. The graphic network was constructed using TCS vers. 1.21 [44]. This method starts by calculating the overall limits of parsimony for the complete data set using a statistic from neutral coalescent theory [45, 46]. Although this method has been used extensively with restriction site and nucleotide sequence data to estimate population level genealogies when divergences are low (intra-specific data) [46, 47], it also proved to be reliable at higher divergences, outperforming parsimony and parsimony with bootstrapping [48]. 

## 3. Results and Discussion 

**Figure 1:**
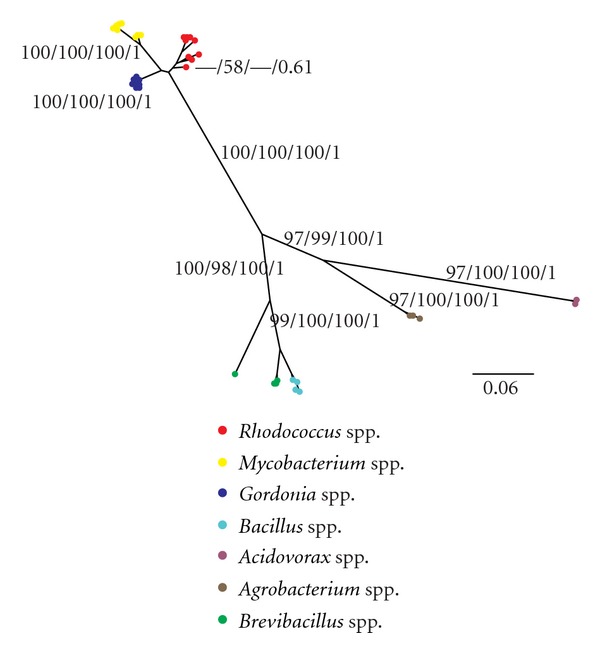
Tree obtained by BA analysis from sequences of the 16S gene. The values on the branches represent bootstrap values of NJ, MV, and MP and posterior probability of the BA analysis.

The BA hypotheses for the 16S gene are shown in Figure 1, which presents the expected pattern of species grouped within their respective genera. The different phylogenetic methods resulted in very similar tree topologies (data not shown) and robust bootstrap values for NJ, ML, MP, and BA posterior probability of the branches. The only exception was the branch containing representatives from the *Rhodococcus* spp. (in red), which showed low bootstrap value in MP (58%), low posterior probability value in BA (0.61), and bootstrap values for NJ and ML < 50%. Although the convergence of results using different phylogenetic methods has been considered good evidence that the correct phylogeny was obtained [49], total genome phylogenies show that different phylogenetic methods can provide incongruent phylogenies [50, 51]. However, the comparison of 16S sequences is still considered a powerful and accepted tool for deducing phylogenetic and evolutionary relationships among bacteria and is routinely used [4, 52–54]. In fact, most of bacteria systematics is based on the topologies generated by this gene [3]. 

**Figure 2:**
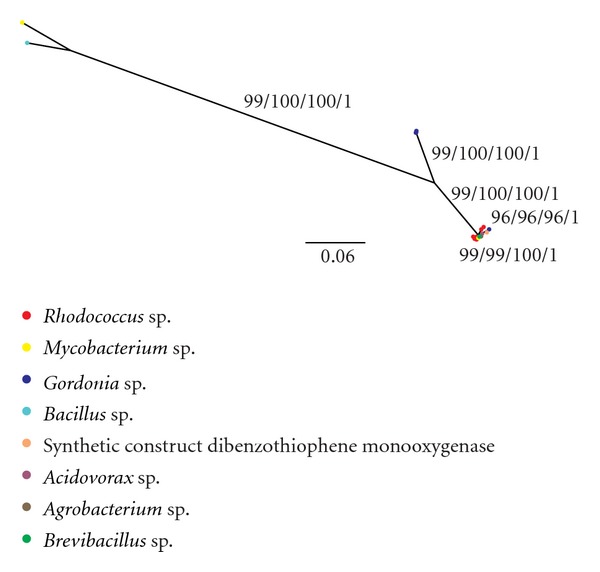
Tree obtained by BA analysis from sequences of the *dsz*C gene. The values on the branches represent bootstrap values of NJ, MV, and MP and posterior probability of the BA analysis.

Figure 2, on the other hand, did not group the species by genera. Instead, this figure presents only three branches: the first includes *Mycobacterium* sp. (strain G3—AB070603.1) and *Bacillus subtilis* (AB076745.1) sequences; the second groups two sequences of *Gordonia alkanivorans *(strain 1 B—AY678116.1 and strain RIPI90A—EU364831.1); the third clusters all remaining sequences belonging to all genera included in this work except for *Bacillus*. It is expected that molecular phylogenies based on single genes lead to apparently conflicting results with alternative branches that present low bootstrap (or posterior probability) values [50]. However, the conflicting topologies shown in Figures 1 and 2 present high bootstrap and posterior probability values in alternative branches, strongly suggesting that the *dszC* was indeed subjected to horizontal transfer events among these bacteria. 

**Figure 3:**
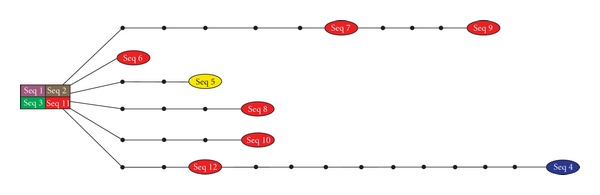
Phylogenetic network estimation of *dsz*C genes that remained grouped after phylogenetic analyses. Seq 1: *Acidovorax delafieldii *(DQ062154.1); Seq 2: *Agrobacterium tumefaciens *(AY960127.1); Seq 3: *Brevibacillus brevis *(DQ062161.1); Seq 4: *Gordonia alkanivorans *(AY714057.1); Seq 5: *Mycobacterium goodii *(EU527978.1); Seq 6: *Rhodococcus erythropolis* (AY714058.1); Seq 7: *Rhodococcus erythropolis* (AY294404.1); Seq 8: *Rhodococcus* sp. (L37363.1); Seq 9: *Rhodococcus* sp. (DQ198086.1); Seq 10: *Rhodococcus* sp. (AY789136.1); Seq 11: *Rhodococcus* sp. (AY278323.1); Seq 12: Synthetic construct dibenzothiophene monooxygenase (EF570783.1).

The phylogenetic network estimation (Figure 3) of the *dsz*C haplotypes of the most specious cluster shown in Figure 2 emphasizes the fact that *Acidovorax delafieldii *(Seq 1), *Agrobacterium tumefaciens *(Seq 2), *Brevibacillus brevis *(Seq 3), and *Rhodococcus* sp. (Seq 11) present identical sequences, which were grouped together as a square within Figure 3, while other haplotypes are displayed as ovals connected to the square by lines with black circles to indicate the maximum number of steps between each pair of haplotypes. 

The results presented here, based solely on GenBank data, provide strong evidence that the *dsz*C gene was horizontally transferred among different evolutionary lineages of bacteria. This evidence is reinforced by the fact that the *dczC* gene is generally found in conjugative plasmids, in the vicinity of insertion sequences, transcribed in the same direction and under the control of a single promoter [35, 37, 55, 56]. Furthermore, another evidence of *dsz*C horizontal transfer is the significant difference of the C+G content of this gene with the C+G content of the entire chromosome of some species studied here (data not shown). 

Our results reinforce the importance of public sequence repositories (such as GenBank), which result from a successful policy of requiring the inclusion of gene sequences in public databases in order to publish any research article containing sequence analyses [57, 58]. In addition to the DNA sequence of each entry, GenBank and other public databases include associated metadata, which provide relevant information about the organism whose sequence is available, generally by linking to the articles with the respective sequence [58]. However, the public databases also contain several molecular sequences submitted by researchers who have not published their results. In these cases, there is neither citation information nor any relevant data about the organisms from which the sequences were made, which in most cases makes the sequences useless for *in silico* works, since diverse knowledge about a given molecular sequence provides an essential first step in developing research hypotheses. 

It is easy to generate new sequences and add them to the GenBank database, which contains about 150 million gene sequences as of February 2012. However, GenBank, along with its INSDC (International Nucleotide Sequence Database Collaboration) partners (EMBL & DDBJ), should be treated not only as archival stores of molecular sequence data (a task at which it has been very successful) but also as a starting point for future studies. In this context, it would be helpful if the process of submitting sequences required a minimum of information about the organism from which the sequences were made, as well as the details of the gene sequenced, in order to substantiate future research. 

In this sense, our study could be improved if flanking DNA sequences of functional genes such as *dsz*C were available in the databases, since we could then evaluate if one set of *dsz* genes is flanked by a particular insertion sequence while another cluster is not. 

Although laboratory data that demonstrate the transfer by conjugation of plasmids containing *dsz* genes or transposition of these genes are scarce, their distribution in bacterial cultures strongly supports the hypothesis that these genes are commonly subject to horizontal transfer in nature as evidenced in the present work. For this reason, we conclude that phylogenetic tools can be useful for inferring horizontal transfer events of functional genes such as *dsz*C. Phylogenetic comparisons with other genes traditionally used for this purpose, such as 16S, can provide good information about evolution and functional gene distribution. 

Lateral gene transfer events provide a venue for bacterial diversification by rearranging existing capabilities. Because bacterial genomes can maintain only a finite amount of information, they are sampling rather than accumulating sequences, counterbalancing gene acquisition with gene loss. As a result, lateral gene transfer can redefine the ecological niche of a microorganism, in effect promoting bacterial speciation [58]. Although a potential result of interspecific recombination is the uncertainty of species boundaries, the increased mixing of genes and the observed phylogenetic inconsistencies show the history of a gene-transfer-mediated diversification of microorganisms.

## Figures and Tables

**Table 1:** Bacteria species names and NCBI accession number of *dsz*C sequences used.

Bacteria species	NCBI accession numbers
*Acidovorax delafieldii*	DQ062154.1
*Agrobacterium tumefaciens*	AY960127.1
*Bacillus subtilis*	AB076745.1
*Mycobacterium* sp. strain G3	AB070603.1
*Brevibacillus brevis*	DQ062161.1
*Gordonia alkanivorans* strain 1B	AY678116.1
*Gordonia alkanivorans*	AY714057.1
*Gordonia alkanivorans* strain RIPI90A	EU364831.1
*Gordonia* sp. strain CYKS2	AY396519.1
*Mycobacterium goodii* strain X7B	EU527978.1
*Rhodococcus erythropolis*	AY714058.1
*Rhodococcus erythropolis*	AY294404.1
*Rhodococcus* sp. strain IGTS8	L37363.1
*Rhodococcus* sp. strain IIPS7	DQ198086.1
*Rhodococcus* sp. strain SDUZAWQ	AY789136.1
*Rhodococcus* sp. strain XP	AY278323.1
Synthetic construct dibenzothiophene monooxygenase (sequence from *Rhodococcus* sp. LY822)	EF570783.1

**Table 2:** Bacteria species names and NCBI accession number of 16S sequences used.

Bacteria species	NCBI accession number
*Acidovorax delafieldii* strain 179	EU730925.1
*Acidovorax delafieldii* strain NBGD35	HQ003420.1
*Agrobacterium tumefaciens* strain NBGD13	HQ003411.1
*Agrobacterium tumefaciens* strain SJ61	GQ140318.1
*Agrobacterium tumefaciens* strain SJ22	GQ140317.1
*Bacillus subtilis* strain DmB55	HQ111354.1
*Bacillus subtilis* strain CCM7	HQ108184.1
*Bacillus subtilis* strain ANctcri3	HQ286641.1
*Bacillus subtilis* strain 13B	HQ335318.1
*Brevibacillus brevis* strain NBGD26	HQ003422.1
*Brevibacillus brevis* strain H2	HM449127.1
*Brevibacillus brevis* strain EIF87	HM480358.1
*Brevibacillus brevis* strain Hot-1	EU327889.1
*Gordonia alkalivorans*	Y18054.1
*Gordonia alkanivorans* strain DSM 44187	AY995556.1
*Gordonia alkanivorans* strain TPR13	EU373422.1
*Gordonia alkanivorans* strain HKI 0136	NR_026488.1
*Gordonia amicalis* strain IEGM	NR_028735.1
*Gordonia amicalis* strain CC-MJ-2a	EU266484.1
*Gordonia amicalis* strain CC-MJ-15b	EU266486.1
*Gordonia amicalis*	AF101418.1
*Mycobacterium avium* strain M214	GU142929.1
*Mycobacterium avium complex* strain 27497	EF611344.1
*Mycobacterium avium* strain ATCC 19698	EF521896.1
*Mycobacterium avium* strain ATCC 25291	EF521895.1
*Mycobacterium avium* strain IWGMT49	EF521892.1
*Mycobacterium goodii*	Y12872.1
*Mycobacterium goodii* strain M069	NR_029341.1
*Mycobacterium goodii* strain X7B	AF513815.1
*Rhodococcus erythropolis *strain ZJB-0910	GU726138.1
*Rhodococcus erythropolis* strain MJ2	GU991529.1
*Rhodococcus erythropolis* strain 13648E	EU741153.1
*Rhodococcus erythropolis *strain e1	EU434599.1
*Rhodococcus* sp. NKCM 2512	AB591806.1
*Rhodococcus* sp. BY44	FR690460.1
*Rhodococcus* sp. ITP08	FR667175.1
*Rhodococcus* sp. SH15	HM590053.1
*Rhodococcus equi* strain ATCC 6939	FJ468344.1
*Rhodococcus erythropolis* strain XP	DQ074453.1

**Table 3:** Software, nucleotide substitution models and criteria used for phylogenetic analysis of 16S and *dsz*C genes in each tested method.

Method	Gene	Nucleotide substitution model	Gamma distribution	Invariable sites proportion
NJ	*dsz*C	Tamura-Nei	0.71	—
	16S	Tamura-Nei	0.69	—
ML	*dsz*C	GTR+G	0.8291	—
	16S	GTR+G+I	0.8125	0.3667
BA	*dsz*C	GTR+G	0.8291	—
	16S	GTR+G+I	0.8126	0.3667
